# The challenges faced in the design, conduct and analysis of surgical randomised controlled trials

**DOI:** 10.1186/1745-6215-10-9

**Published:** 2009-02-06

**Authors:** Jonathan A Cook

**Affiliations:** 1 Health Services Research Unit, University Of Aberdeen, Health Sciences Building, Foresterhill, Aberdeen, Scotland, AB25 2ZD, UK; 2 Clinical Epidemiology Program, Ottawa Health Research Institute, 1053 Carling Avenue, Ottawa, Ontario, K1Y 4E9, Canada

## Abstract

Randomised evaluations of surgical interventions are rare; some interventions have been widely adopted without rigorous evaluation. Unlike other medical areas, the randomised controlled trial (RCT) design has not become the default study design for the evaluation of surgical interventions. Surgical trials are difficult to successfully undertake and pose particular practical and methodological challenges. However, RCTs have played a role in the assessment of surgical innovations and there is scope and need for greater use. This article will consider the design, conduct and analysis of an RCT of a surgical intervention. The issues will be reviewed under three headings: the timing of the evaluation, defining the research question and trial design issues. Recommendations on the conduct of future surgical RCTs are made. Collaboration between research and surgical communities is needed to address the distinct issues raised by the assessment of surgical interventions and enable the conduct of appropriate and well-designed trials.

## Background

The promotion of randomised controlled trials (RCTs) to evaluate surgical interventions was once colourfully suggested to be the 'fifth horseman of an apocalyptical surgical fundamentalism' [1]. While the value of the RCT design has been more readily accepted by others, it has not become the default study design for the evaluation of new surgical interventions [2-4]. This article will consider the challenges to successfully conducting an RCT evaluation of a surgical intervention. Surgical interventions can be defined as those which involve physically changing body tissues and organs through manual operation such as cutting, abrading, suturing or the use of lasers. It should be noted that some authors use a wider definition of surgical trials [3] which includes trials in a surgical context, where surgery is involved but is not one of the interventions under evaluation, for example a placebo-controlled trial of ibuprofen for pain and disability relief after hip replacement surgery [5]. While many of the issues raised below have relevance for such trials, they have been much more readily conducted and do not face the same challenges.

Since the epochal streptomycin trial in the 1940s, the RCT design has been applied widely [6]. As understanding of the nature of study design and of the influence of bias has grown, the RCT design has for many become the 'gold standard' of evaluation, the standard against which others are compared. Through random allocation of participants, equally distributed groups are formed in a RCT. This allows for any difference between treatment groups to be confidently inferred to be due to the treatments themselves and not any other factor, known or unknown to be related to outcome. Where equipoise exists, it can be argued from an ethical point of view that every participant is guaranteed a (random) chance of receiving the best treatment. With the requirements of regulatory bodies such as the Food and Drug Administration in the USA, the RCT has been not only expected but mandated in the pharmaceutical area. Through the strong promotion of Chalmers amongst others [7] and the evidence-based medicine movement in general, it is common for new interventions to be evaluated in an RCT context. While recognising RCTs as the 'gold standard', it has been suggested that the design has a more limited role in assessing surgery than for drug interventions [2,8,9]. However, findings from alternative (non-randomised) study designs cannot be given the same confidence, due to the substantial risk of the introduction of bias. Comparisons of randomised and non-randomised studies have shown that the results can be divergent, in direction as well as magnitude [10]. While many surgeons accept the need in principle for RCTs, they struggle to reconcile their personal involvement with their surgical experience.

Randomised comparisons of surgical interventions have been performed for many years [7]. A number of surgical interventions have been shown to be ineffective and later discarded, in some cases following randomised comparisons [11]. Internal mammary artery ligation was a popular surgical procedure until two small RCTs, some 20 years after the intervention was proposed, reported no benefit over placebo surgery. There is, however, some evidence that the growth in the number of RCTs being conducted in surgery has stagnated and fallen behind other clinical areas [8,12]. A review of one surgical journal found an increase in the number of RCTs from 1990 to 2000 [13]. However, only 3.4% of all articles in leading surgical journals were RCTs [14]. Furthermore, of the RCTs published in these journals during a ten-year period, less than one half of the RCTs were a randomised comparison of a surgical intervention against an alternative. The contrast with other areas can be seen by comparing audits of the evidence base for clinical practice in internal medicine and several surgical specialties. Whereas one half of interventions in internal medicine were judged to be based upon evidence from RCTs, two surgery audits reported a quarter or less [15-17].

While there are genuine challenges to conducting surgical trials, concerns about the quality of surgical trials are often more general and more readily addressable. Reviews of urological and orthopaedic trauma trials, which recognised the difficulties inherent in conducting surgical trials, found poor compliance with the consort statement for reporting RCTs, echoing the findings of an earlier review, and may reflect generally poorer trial methodology [18-20]. A lack of understanding about RCTs in surgical communities and the need for better epidemiological and statistical training of surgeons have been noted [3,21]. Historical and ethos-related reasons as to why the surgical community has been slow to change have been suggested [4].

The difficulties of conducting an RCT of a surgical intervention have long been recognised [21-25]. Surgical RCTs may have suffered as understanding of bias has grown, as the maximum safeguards against bias are difficult to achieve, particularly compared with pure pharmaceutical treatments. A review of surgical operative questions in the gastrointestinal specialty suggested that while there was, at least in principle, substantially more scope for RCTs, there were many questions where an RCT was judged not to be feasible [26]. A number of barriers to conducting surgical RCTs have been suggested [2,3,9,27,28]. Stirrat and colleagues produced a helpful summary of the issues involved and some suggestions to overcome them [9]. More recently, two other groups have done similarly [3,28]. An overview of the issues and possible solutions will be reviewed under three headings: the timing of the evaluation, defining the research question and trial design issues. It should be noted that a systematic review and corresponding meta-analysis can play an invaluable role in trial design.

Broadly, surgical trials can be classified into three generic types: exploratory, explanatory and pragmatic trials. Exploratory trials allow early assessment of new interventions. An explanatory trial seeks to assess whether the intervention can work under favourable conditions. In contrast, a pragmatic trial seeks to inform clinical decision making by evaluating an intervention in a realistic clinical setting. General characteristics of the three types are shown in Table 1, though in practice a trial may have some characteristics that reflect more than one type or which defy easy categorisation. In particular, a continuum between explanatory and pragmatic trials exists [29]. It should be noted that the evaluation of surgery, by its complex nature, naturally fits a more pragmatic framework. By adopting a generally pragmatic approach, the impact of many of the trial design issues is diluted. For example, legitimate variations in surgical/centre practice or some patients not receiving surgery as initially planned can be incorporated into the evaluation, as opposed to attempting to preclude them from occurring. Such an approach evaluates a realistic management strategy involving the surgical intervention. As a consequence, the findings from a trial with this approach will be more widely applicable.

**Table 1:** Characteristics of three generic forms of surgical trials

Study characteristic	Exploratory trial	Explanatory trial	Pragmatic trial
Aim of evaluation	To explore the impact of the intervention	To assess whether the intervention is efficacious	To assess whether the intervention is effective
Patient population	Initially those presumed to be most likely to benefit, though later modification is allowed	Narrow inclusion criteria of patients expected to be most suited to treatment	Broad inclusion criteria reflecting variations in clinical practice between centres
Surgical setting	Surgeon(s) with substantial generic surgical expertise	Surgeon(s) with expertise in the intervention under evaluation	Surgeons from multiple centres representing different levels of expertise
Intervention definition	Freedom to develop and refine	Tight definition and strictly controlled	Broader definition incorporating variations which reflect clinical practice
Outcomes of main focus	Surgical process and short-term clinical outcomes	Short-term (sometimes surrogate) clinical outcomes	Longer-term clinical and patient-reported outcomes (such as quality of life measures)

## The timing of the evaluation

### When to assess?

Unlike the four phases of clinical trials which map out the evaluation of pharmaceutical interventions, the evaluation of surgical interventions has been sporadic, with the timing or existence of a randomised evaluation uncertain. The absence of a strong regulatory framework has resulted in the proliferation of surgical innovation on the basis of limited and weak scientific evidence [3,30,31]. The classic example of recent times has been the case of laparoscopic cholecystectomy, where adoption was rapid, uncontrolled and haphazard. RCT evaluation occurred late, after much of the surgical community had already become convinced of the worth of the technique [3,31]. Little, if any, randomised evidence is usually available upon which to base recommendations on safety and efficacy for new interventional (including surgical) interventions [32]. Most surgical RCTs, as was the case for laparoscopic cholecystectomy, are akin to phase III pharmaceutical trials.

However, there is a role for early and appropriate RCTs to allow rigorous and timely assessment. Indeed, in Chalmers' seminal work on randomisation from the first patient, he used a surgical example where randomisation from the first handful of cases was undertaken [7]. Chalmers argued strongly for early RCT assessment of surgical innovations, arguing from ethical reasoning of guaranteeing a patient's right to a (random) chance of receiving the best treatment and also from the difficulty of fully informing patients in the early cases of development [22]. Others argue the need for a non-randomised study in a developmental phase due to procedural refinement and associated learning effects [2,3]. However, the complementary value of an early and later randomised comparison has been highlighted [33]. An early evaluation could take the form of an exploratory trial. Tracker trials have been proposed as a flexible framework which encompasses the different stages of evaluating a new intervention in a single study. Under such an approach, randomisation can take place early, with procedural refinements and new intervention options incorporated into the study by allowing changes in the randomisation strategy. These developments are 'tracked' and assessed in subsequent statistical analyses [34]. Once the technology and treatment options have stabilised, a conventional phase III RCT could be mounted within this framework as a continuation of the tracker trial. The UK Endovascular Aneurysm Repair trials adopted some features of this concept [28]. Despite the scientific merits of such a comprehensive approach, to date no fully fledged tracker trials have been undertaken.

The difficult balancing act of waiting until development has settled, where early results can lead to the loss of equipoise, has long been recognised [7]. The challenge has been distilled into what has become known as Buxton's law: 'It is always too early [for rigorous evaluation] until suddenly it's too late' [35]. Large surgical trials, once major technology has stabilised, have become more common where equipoise still exists. Such trials will be dependent on the persistence of equipoise (patient and surgeon) and may fail to recruit as hoped [2]. Commercial influences can come into play. In general, early and rigorous randomised evaluation would seem to be the exception rather than the rule.

### Funding

An important driver of the timing of an evaluation has been availability of funding. Anecdotally it has been suggested that surgical trials are often unfairly compared with the expectation of a drug trial, and if a 'double-blind' placebo-controlled study is the standard to which it is held, then a surgical trial will often be considered inferior. In contrast to pharmaceutical treatments, there is generally little commercial incentive for a company to fund randomised surgical trials. The need for national funding bodies to cater for surgical trials and explicitly recognise the challenges has been noted [3]. A review of applications to the National Institutes of Health in the USA showed that though surgical applications had grown over the last 20 years it was at a lower rate than the overall increase. The surgical success rate was also slightly lower [36]. Infrastructure funding to support clinical trials units that specialise in the evaluation of surgical trials would increase capacity. While sufficient funding for rigorous randomised evaluation of every surgical innovation may not be realistic [4], major innovations should be subject to rigorous evaluation and appropriate funding is needed.

## Defining the Research Question

### Choice of comparator

In defining the research question, the most important decision is the choice of comparator. Sometimes surgical intervention may be the only curative treatment. Comparison of surgery against no active treatment or watchful waiting may be appropriate where the condition is not acute. The possibility of using placebo surgery will be considered later. A key consideration will be the ability to recruit; this has been noted as being difficult, particularly for medical versus surgical comparisons [9,37,38]. A number of variations in randomisation strategies have been adopted to combat the difficulty in recruiting surgeons and participants. Traditionally, a randomised trial will involve the randomisation between two treatments whereby the randomised treatment will be administered by the same clinician. Where the treatments are from different clinical specialties, such as medical versus surgical trials, this will not be case. However, where two surgical treatments are being compared, it has been common for both interventions to be performed by participating surgeons. If the difference between interventions is minor, this may not be difficult to achieve. However, where differences are more pronounced this can be problematic, as surgeons may routinely only perform one or the other intervention. Additionally, while the surgical community can be in equipoise, individual surgeons may have strong preferences. In light of these concerns, the use of expertise-based randomised trials has been proposed as an alternative design, where participants are randomised to surgeons with expertise in the allocated intervention [25,39]. While some surgeons may perform both interventions, there is no requirement to do so. Where expertise for both surgical interventions is available in a centre, this would seem a convenient solution. However, in a multicentre trial, if some centres only have expertise in one intervention, centre differences could lead to bias. More generally, an expertise-based trial may not necessarily produce a result generalisable to the whole surgical community, due to the make up of the participating surgeons and centres.

Patient preferences are likely to be prominent when the treatments differ greatly, for example in medical versus surgical trials. Conducting parallel preference arms alongside the randomised arms, often called the comprehensive cohort design, allows collection of data from participants who are unwilling to be randomised and addresses concerns about generalisability due to low inclusion rates [40]. However, the cost and limited added benefit has resulted in limited usage. Offering a new intervention only within the context of an RCT may lift participation rates, though evidence for this is limited and this will only be possible if widespread adoption has not occurred [41]. The randomisation by consent design (Zelen's design) has been proposed to address the difficulty of gaining informed consent [42]. Under this approach, eligible patients are randomised and consent is sought post-randomisation only for those receiving the new interventions, with consent to standard care presumed unnecessary. However, most agree this is not ethical and only moves the problems downstream [28]. Insights into the consenting process may enable increased participation while maintaining informed choice [43].

Where three or more treatment options are under evaluation, along with the option of randomisation between all treatments additional options which involve randomisation between subsets of interventions can be offered. In principle this will allow surgeons who feel unable to randomise between all treatments to participate in a stratum with which they feel comfortable. For example, the STARS trial involved an evaluation of three surgical interventions: reduction and fixation, bipolar hemiarthroplasty and total arthroplasty [44]. Participating surgeons were offered two choices: to randomise between all three interventions, or only between reduction and fixation, and hemiarthroplasty. As noted by Lilford and colleagues, evidence from non-randomised (indirect) comparisons may account for centre differences [28].

### Intervention definition and inclusion criteria

**Figure 1:**
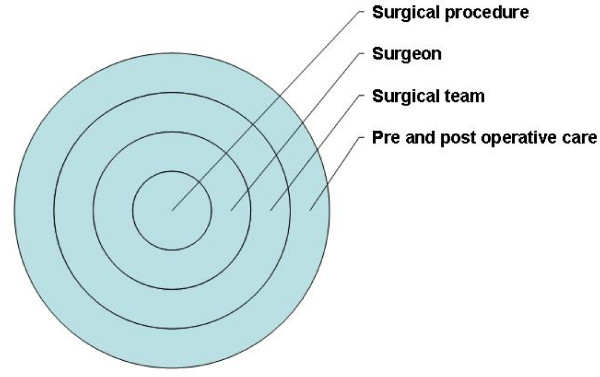
**Main constituent elements of a surgical intervention**.

Trials which evaluate a surgical intervention will come under scrutiny regarding the patient population and the integrity of the treatment groups [3,21]. A surgical intervention can be viewed as a complex intervention [45]. Figure 1 illustrates the main constituent elements. In addition to the interaction of surgeon and procedure, the wider context of the surgical team and pre-operative and post-operative care are important. Clinically unnecessary delays in receiving treatment, for example due to waiting times in a national health service context, can also influence outcome [47]. A relatively loose definition of the intervention while maintaining the coherence of the treatment will best reflect practice. A trial comparing hysterectomy versus medical management left the type and route of the intervention performed at the discretion of the gynaecologist [38]. In general, broad inclusion criteria will aid applicability of the results and is to be favoured. In the Spine Patient Outcome Research Trial, an evaluation of surgical versus non-surgical care for lumbar disc herniation, only 60% in the surgical arm received surgery while 45% in the non-surgical arm received surgery, highlighting that substantial non-compliance can occur in some situations [47]. A more explanatory approach with a tighter definition of participant inclusion and intervention can be warranted. Whatever the focus of the trial, clear reporting of how the treatment was defined and compliance is need [48].

### Surgical learning curve

A frequent criticism of surgical RCTs, particularly when evaluating a new innovation against a standard intervention, is that the comparison may be inherently 'unfair' due to an imbalance in expertise. When a randomised comparison of two forms of gastric cancer surgery, contrary to previous studies, found a higher rate of complications for the newer D2 surgery, it was argued that the surgeons were still learning in the trial and the 'true' performance of D2 surgery was not represented [33,49]. This underlying phenomenon of an improvement in performance over time is commonly referred to as a 'learning curve'. It has been described as the most intractable of the obstacles to conducting surgical randomised trials [9,50]. Scenarios in which a surgical learning curve effect could lead to an incorrect conclusion have been outlined [33].

Two distinct, though related, learning curves can be defined. First is a community or technology learning curve, related to refinement of the new intervention (both technology and technique). Second is the personal learning of individual surgeons which, though impacted by the former, is mainly driven by their personal aptitude, training and surgical experience, although factors such as the make-up of the surgical team and centre policies also contribute. Training prepares a surgeon for undertaking a surgical intervention; however, the extent to which it can replace real case experience is unclear, and it would seem that some expertise can only be acquired in the real-life setting. By adopting a late evaluation strategy, the first learning curve will be overcome whereas the latter will remain, though its impact lessened through the gains of the former.

Two main options exist for controlling the impact of a learning curve in the context of a clinical trial: a design and an analysis approach. In terms of design, entry criteria for eligible participating surgeons can stipulate characteristics such as the number of procedures performed, minimum professional level and training received. For example, a trial comparing open and laparoscopic mesh repair of inguinal hernias required, prior to participation, surgeons to have performed 10 laparoscopic interventions and to demonstrate their competence by undertaking with supervision another five interventions [51]. Adoption of an expertise-based design for surgical trials would similarly require some mechanism to identify those with sufficient 'expertise' to undertake a particular surgical intervention.

In principle, a statistical assessment of the learning curve would be preferable as it would allow the impact of the learning curve on outcome to be quantified. Until recently this has received relatively little attention, though the advent of minimally invasive interventions has lead to increased interest. Ramsay *et al *reviewed both the assessment of learning curves in health care and also the statistical methods used to assess the learning curve across disciplines [35]. In general, studies have focussed upon process measures such as intervention time, blood loss, hospital stay and complication rates, with little focus upon more patient-centred outcomes. Generally, the statistical methods used have been inadequate, though more sophisticated methods are available which can be used. A hierarchical model was used for a large trial assessing suturing material and technique for perineal repair [33]. A post-hoc analysis in a trial of over 2000 participants, which compared laparoscopic and open tension-free mesh repairs of inguinal hernias, found a reduction in recurrence in the laparoscopic group with surgeon expertise [52]. Due to the large data requirement for analysis, data on intervention learning is most likely to come, if available, from large non-randomised studies which can be incorporated into corresponding economic evaluations. A reduction in the five-year recurrence rate of prostate cancer after radical prostatectomy was observed in a cohort of over 7000. This reduction persisted, even after a surgeon had performed a large number of procedures [53].

Surgical trials should report explicitly and informatively on the prior expertise of the participating surgeons [54]. Failure to do so will open the trial up to criticism. A survey of participating surgeons could provide valuable information. Justification, with reference to the research question, should be made for how the existence of a learning curve is addressed. A truly pragmatic trial should seek to include expertise levels which are representative of the surgical community in which the intervention will be used. This may require a relatively low level of procedural expertise. A trial conducted by the most experienced surgeons in the high-caseload centres may not transfer readily to other settings. Conversely, an explanatory trial could seek to largely exclude learning by only including highly experienced surgeons. An early evaluation could conduct exploratory statistical analysis of the learning curve [33].

## Trial Design issues

### Blinding

Blinding – the process of withholding knowledge of the allocated intervention – is a key consideration when designing RCTs. Where successfully achieved, it is recognised as playing an important role in preventing the introduction of bias. It can also aid compliance and retention of participants [55]. Blinding can be applied to participants, investigators and the outcome assessors of a trial. Where interventional treatments are involved, blinding poses a greater challenge than for pharmaceutical-based treatments [56]. The extent to which blinding is feasible or necessary will depend upon the nature of the interventions and also the outcome under consideration.

Blinding of the surgeon for evaluations of surgical techniques will be impossible. Outcome assessors can, and preferably should, be blinded. Surgeons blinded to the allocation could assess clinical outcomes [57]. A review of RCTs in a leading orthopaedic journal showed large bias due to unblinded outcome assessors [58]. However, blinding is not commonly undertaken in surgical trials [59]. This is probably partly due to the standard care pathway, where patients commonly return to the surgeon who performed their surgery for re-assessment. It may also be due to the mistaken belief that because the operating surgeon cannot be blinded, no blinding is worthwhile or achievable.

Blinding of participants in surgical trials can often be achieved. Where two similar surgical interventions are compared, such as the comparison of two forms of laparoscopic hernia repair, blinding can readily be undertaken. Where surgical interventions differ significantly, blinding of the participant is more problematic, though may still be possible. For example, a trial comparison of laparoscopic and open cholecystectomy used the same dressing for both interventions to allow blinding of participants and caregivers [60]. Where a surgical intervention is compared with a medical intervention, blinding of the participant will not be feasible without some concurrent sham intervention, which is unlikely to be considered ethical.

The use of placebo or sham surgery, while remaining controversial, has been achieved in a number of cases [61]. A placebo-controlled evaluation of arthroscopic surgery for osteoarthritis of the knee found no benefit over the sham intervention [62]. However, the scope for placebo surgery is limited to cases where the risk inherent in the placebo surgery can be balanced against the uncertainty of the value of the active surgery and its potential harm. Where a genuine treatment alternative is available, surgical or not, placebo surgery would seem neither ethical nor desirable.

### Mechanism and timing of randomisation

Concealment of future allocation is important. It prevents manipulation, conscious or not, of group allocation, protecting the merit of randomisation of balanced groups at baseline. Where funding permits, an automated telephone randomisation service run by a trial office is preferable. Whatever the mechanism of randomisation, third-party control is highly desirable. Ideally, treatment will be received soon after randomisation. Randomisation can be performed in the operating theatre where participating surgeons undertake both interventions [51]. Where allocated interventions are performed by different clinicians, as in an expertise-based trial or medical versus surgical trials, randomisation will need to be earlier. For multicentre trials stratification is important, as even where the most technical intervention is involved variation between centres can be substantial. Minimisation, a numerical method which allocates a participant to the group which maximises the equality between groups, can be used to ensure equally distributed prognostic variables where the trial size is small by including factors known or believed to strongly influence treatment outcome [63].

### Outcomes

A variety of outcomes are needed in surgical trials: surgical and clinical outcomes, patient-reported outcomes such as quality of life measures, and also economic outcomes. Outcomes related to the surgical process such as complications and other short-term outcomes have received much focus, particularly for new and complex interventions. However, longer-term outcomes will for many situations be more important [9]. Meta-analysts can be confronted with a plethora of outcomes, with outcomes such as recurrence or complications having varied definitions. Consensus on which outcomes are important and how they should be measured is needed. Independent assessment of outcome is highly desirable. Some outcomes may be prohibitively rare to base trial sample-size calculations upon. Data, on which to base sample-size calculations, can be limited. Where substantial uncertainty exists, an adaptive design approach could be used. In principle, all surgical trials will be subject to a clustering effect and sample sizes should be inflated to account for this, though the impact is uncertain [64].

### Data collection and monitoring of surgical compliance

Often, surgical details can only feasibly be collected through self-report by the surgeon. Independent data collection, for example through a study nurse, is preferable. Adjudication of outcome events by independent and blinded review of clinical data adds methodological rigour. Intra-operative photographic evidence of the procedure has been used to allow independent assessment of surgical adherence to the protocol [65]. Short-term post-surgery data is useful for assessing recovery. Collection of patient-reported outcomes, for example through postal questionnaires, provides an important perspective [44]. A meeting of surgeon investigators at the start of a trial along with site visits/teleconferences during the study can help clarify and reaffirm the trial protocol and ensure consistent data reporting.

### Statistical Analysis

Outcomes following procedures performed by the same surgeon (or centre) will tend to be more similar than those performed by another. Statistical methods which take account of this inherent structure in surgical trials do exist and should be used in the analysis where studies are of sufficient size [64]. Subgroups of possible treatment modifiers should be considered and analysis based upon a test for an interaction [66]. For a variety of reasons, participants randomised to a surgical intervention may not receive their allocated intervention. Cross-over between interventions is not uncommon for surgical trials and can happen prior, during or post the scheduled intervention and can be asymmetric [47]. RCTs should be analysed by grouping participants according to their allocated treatment irrespective of subsequent non-compliance. In some situations an additional analysis, excluding non-compliant cases ('per-protocol' analysis) or using statistical methods which allow a compliance-based analysis, may be useful to provide a more realistic estimate of the compliant setting, especially for more explanatory trials [67]. Post-randomisation exclusion may be appropriate where new clinical information reveals, after randomisation, the participant not to be suitable for treatment. However, exclusions threaten the value of randomisation, and reasons should be specified prior to commencement of randomisation [68].

## Conclusion

RCTs have played a role in the assessment of surgical innovations and there is scope and need for greater use. Surgical trials are difficult to successfully undertake and pose particular practical and methodological challenges. However, the inherent value of a well-conducted RCT should not be overlooked. Collaboration between surgical and research communities is needed to enable the conduct of appropriate and well-designed trials. Three recommendations for the future conduct of RCTs in surgery are as follows:

• Innovations should be stratified according to their potential relevance to practice. The highest impact innovations should be prioritised for RCT evaluation where such an evaluation is ethical and feasible. Surgical communities could take the lead in this process with input from the research community and funding bodies to achieve this goal.

• Pragmatic trials have been under-represented in surgery and this approach should be adopted more widely.

• Exploratory trials (and by extension, tracker trials) can play a role in the early evaluation of surgical innovations and should be more readily considered.

## Competing interests

The author declares that he has no competing interests.

## Authors' contributions

JC wrote the manuscript and has read and approved the final version.
